# A case report on a rare presentation of adrenal myelolipoma with low-impact traumatic haemorrhage and the challenges of conservative management

**DOI:** 10.1093/jscr/rjac554

**Published:** 2022-12-07

**Authors:** Bronte Paice, Taiwo Oyebola, Kathryn Ball, Shaun Alexander, Benedict Sherwood

**Affiliations:** Department of Urology, Nottingham University Hospitals, Nottingham, UK; Department of Urology, University Hospitals of Derby and Burton, Derby, UK; Department of Urology, University Hospitals of Derby and Burton, Derby, UK; Foundation Training, United Lincolnshire Hospitals, Lincoln, UK; Department of Urology, Nottingham University Hospitals, Nottingham, UK

## Abstract

Adrenal myelolipomas are rare, hormonally silent, adipose and myeloid-containing lesions that are mostly asymptomatic. If they do present it is usually with mass-related flank pain or spontaneous haemorrhage. A 55-year-old female presented with right flank pain after a fall from a static pushbike. Computer tomography identified a large adrenal lesion with surrounding acute retroperitoneal haemorrhage. A conservative approach to treatment was decided on as the patient remained haemodynamically stable. The patient developed a pulmonary embolism during the time of conservative management and therefore had to be anticoagulated with close monitoring. Outpatient surveillance imaging was reassuring, hormonal screening was negative and biopsy confirmed myelolipoma. We report a rare presentation of adrenal myelolipoma with the sequelae of haemorrhage from low-impact trauma and the challenges of conservative management.

## INTRODUCTION

Adrenal myelolipomas (AMLs) are benign and hormonally silent lesions of the adrenal interstitium. AMLs are rare with an autopsy prevalence of 0.08–0.2% [1]. They are mainly composed of mature adipose and myeloid tissues. Extramedullary haemopoiesis within the adrenal gland is a physiological process in the developing foetus, but only persists into adult years pathologically [2]. Lesions with higher content of myeloid tissue have a higher disposition to bleeding. AMLs are mostly asymptomatic, but may present with flank pain and spontaneous haemorrhage when larger (>6 cm) [3]. They are slow-growing and therefore on initial imaging if <4 cm in size, the European Society of Endocrinology (ESE) recommends that these patients will not need further follow-up unless become symptomatic. In light of AMLs rarely becoming symptomatic, they are often referred to as one of the ‘incidentalomas’ and are commonly first identified on imaging indicated for another clinical question [4]. Due to the high lipid content, AMLs have characteristic Computer Tomography (CT) findings; well circumcised, heterogeneous area of the adrenal gland with attenuations < 0 HU pre-contrast [5].

## CASE REPORT

**Figure 1:**
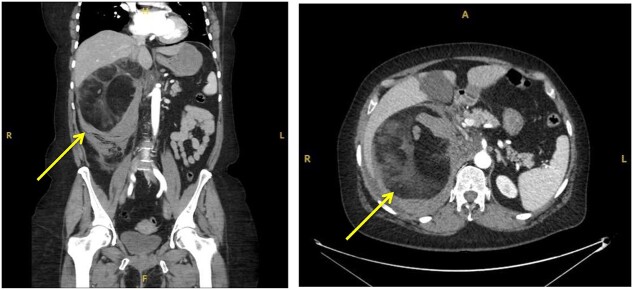
Coronal (**a**) and transverse (**b**) sections of initial CT imaging with contrast showing large lesion with heterogeneous hypodense areas with surrounding haematoma (arrowed).

A 55-year-old Caucasian female presented to the emergency department (ED) after falling from sitting on a static pushbike onto her right flank. She immediately incurred right flank pain with nil other symptoms at time of the event. Her medical history was insignificant and she did not take any regular medications. Blood tests on admission indicated there may be an intra-abdominal injury due to raised lactate of 3 and a blood pH of 7.304. An urgent CT abdomen and pelvis (CTAP) with contrast was performed which showed a large (15.2×13.8 cm), predominantly fat containing mass in the right suprarenal region causing displacement of the right kidney and pancreas, with surrounding hyperdense fluid suggestive of acute retroperitoneal haemorrhage (Fig. 1).

**Figure 2:**
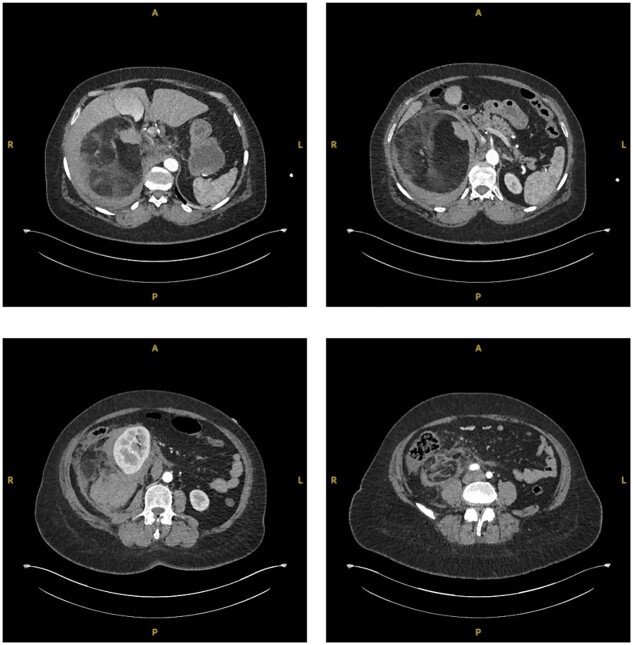
Sequential transverse sections of CTAP in arterial phase showing extent of lesion down to level of right Ilium bone.

The patient was subsequently transferred over to the local specialty Urology Centre. Here the patient became tachycardic and required supplemental oxygen. A suspicion of increasing blood loss or a pulmonary embolism (PE) was raised. A repeat CTAP in arterial and venous phases along with a CT pulmonary angiography (CTPA) was conducted. The CTAP again showed a large right-sided retroperitoneal mass, now described as mixed fat and soft tissue densities characteristic of AML. The surrounding retroperitoneal bleeding was thought to have increased in size, indicating likely active haemorrhage (Fig. 2). CTPA showed no PE.

The patients’ ongoing oxygen requirement along with the CT findings of increasing size of haematoma resulted in admission to the high-dependency unit (HDU) for high-flow oxygen administration and close monitor for signs of haemodynamic instability. Throughout the patient remained hameodynamically stable and did not require transfusion (Table 1).

**Table 1:** Blood test results

	Day 0	Day 1	Day 2	Day 3	Day 4	Day 5	Day 6	Day 7
Hb (g/L)	140	123	94	87	84	95	100	93
eGFR (mL/min)	83	>90	>90	>90	>90	>90	>90	>90

The patient was stepped-down from HDU after 4 days. Thereafter, management options were explored including interventional radiological embolization of feeding artery, surgical exploration +/− adrenalectomy and conservative bed rest with empirical broad-spectrum antibiotics and tranexamic acid. As the patient remained stable and no appropriate target vessels were identified on imaging for embolization, a conservative management approach was deemed most suitable in this case.

After 7 days, the patient was discharged with advice to rest with minimal exertion for the following 6 weeks. Low-molecular weight heparin was not prescribed due to risk of worsening haematoma. An outpatient CTAP was planned for 2 weeks post-discharge.

**Figure 3:**
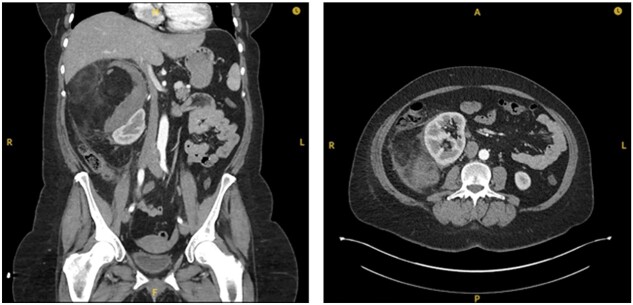
Interval CT imaging showing decrease in size of lesion and surrounding haematoma.

On interval CT scanning, the adrenal lesion was shown to have decreased in size (14.3×13.2 cm), likely due to reduction of intralesional haematoma, and no active haemorrhage identified (Fig. 3). There was an incidental finding of filling defects in the right lower lobe pulmonary artery. The patient was therefore re-admitted to work-up for PE. The patient was asymptomatic of venous thromboembolism. A CTPA identified bilateral segmental and sub-segmental pulmonary vessel filling defects, with no evidence of right heart strain. This therefore warranted commencement of anticoagulation; however, the patient remained at risk of re-haemorrhage of the adrenal lesion. On discussion with different relevant specialties and after counselling the patient on the risks and benefits, it was decided to commence on treatment dose Enoxaparin (low-molecular weight heparin). Due to her higher propensity of haemorrhage, the patient remained as an inpatient for close observation of signs of haemodynamic compromise. The patient remained well on Enoxaparin and was eventually discharged.

After identifying a lesion on imaging, the ESE advises that assessment of hormone excess should be analysed, including dexamethasone suppression test, plasma-free metanephrines or urinary fractionated metanephrines [6]. These were performed in the outpatient setting for our patient. As suspected, these biochemical tests were negative confirming a hormonally silent lesion (Table 2).

**Table 2:** Dexamethasone suppression test results

	Baseline	Suppression
ACTH (ng/L)	12	<5
Cortisol (nmol/L)	303	34

To further confirm a benign lesion, an ultrasound-guided biopsy of the adrenal lesion was performed which gave a histological confirmation of myelolipoma.

## DISCUSSION

AMLs are rare, benign, slow growing, non-secretory and biochemically inert lesion of the adrenal gland. They are composed of adipose and haematopoietic cells of myeloid tissue. They are most commonly asymptomatic and diagnosed primarily incidentally on cross sectional imaging in up to 90% of cases i.e. adrenal incidentaloma [7]. The adrenals deep abdominal location means they are protected from trauma and symptomatic bleeding of AMLs is therefore mostly seen spontaneously and in larger lesions of 10–12 cm [8]. We report a rare case of traumatic haemorrhage of AML on low-impact trauma and complications incurring on conservative management. We highlight that AMLs are at a disposition to bleed on minimal impact trauma when large enough in the retroperitoneal space and how it is important to investigate patients based on symptoms rather than mechanism of injury on a case-by-case basis.
